# Targeting of CCN2 suppresses tumor progression and improves chemo-sensitivity in urothelial bladder cancer

**DOI:** 10.18632/oncotarget.19987

**Published:** 2017-08-07

**Authors:** Xiaojing Wang, Tianyuan Xu, Fengbin Gao, Hongchao He, Yu Zhu, Zhoujun Shen

**Affiliations:** ^1^ Department of Urology, Ruijin Hospital, School of Medicine, Shanghai Jiaotong University, Shanghai, China; ^2^ Department of Urology, Huashan Hospital, Fudan University, Shanghai, China

**Keywords:** CCN2, bladder cancer, chemotherapy, mitomycin C

## Abstract

Urothelial bladder cancer (UBC) is the most common urinary neoplasm in China. CCN family protein 2 (CCN2), a cysteine-rich matricellular protein, is abnormally expressed in several cancer types and involved in tumor progression or chemo-resistance. However, detailed expression patterns and effects of CCN2 in UBC still remain unknown. We found that down-regulation of CCN2 suppressed proliferation, migration and invasion of UBC cells *in vitro* and targeting of CCN2 decelerated xenograft growth *in vivo*. When treated with mitomycin C (MMC), CCN2-scilencing UBC cells showed lower survival and higher apoptotic rates and these effects were probably mediated via inactivation of Akt and Erk pathways. We also demonstrated the clinical significance of CCN2 expression, which was higher in UBC tissues and associated with advanced tumor stage and high pathologic grade. Taken together, our data suggest that CCN2 is an oncogene in UBC and might serve as a matricellular target for improving chemotherapeutic efficacy.

## INTRODUCTION

Urothelial bladder cancer (UBC) is a common urinary malignancy. In 2012, about 429,800 new cases and 165,100 deaths arising from UBC occur worldwide [1]. In the United States, UBC is estimated that to be the second most frequent genitourinary tract cancer and the fourth most common cancer in male in 2017 [2]. At initial diagnosis, approximately 80% of UBC cases present with the superficial form, non-muscle invasive bladder cancer (NMIBC), which is routinely treated by transurethral resection of bladder tumor (TURBT). Other UBC patients suffer from muscle invasive bladder cancer (MIBC), which could also be derived from superficial NMIBC lesions, and these cases generally received radical cystectomy. As a result of inherent tumor biological properties, UBC surgeries are followed by high possibilities of disease recurrence, progression or cancer-specific mortality. Chemotherapy is the most important adjuvant treatment for both NMIBC and MIBC. However, after initially favorable response, inferior chemo-sensitivity or even chemo-resistance may arise due to alterations in drug metabolism, DNA repair or apoptotic pathways [3, 4]. Hence, relevant studies are urgently needed to further reveal mechanisms of UBC development and optimize current therapeutic strategies.

CCN family protein 2 (CCN2), also known as connective tissue growth factor (CTGF), belongs to the CCN family of cysteine-rich secretory matricellular proteins. By interacting with extracellular matrix (ECM) or cell receptors (e.g., integrin), CCN2 controls a number of biological processes including cell mitogenesis, differentiation, adhesion, movement and angiogenesis [5]. In many cancer types, such as osteosarcoma, melanoma and breast cancer, CCN2 is abnormally expressed and involved in tumor progression or chemo-resistance [6]. Based on microarray data of UBC specimens, Fang et al [7] reported the differential expression of CCN2 among different tumor stages and implied its significance in UBC development. However, the detailed expression patterns and effects of CCN2 in human UBC still remain unknown.

To this aim, we conducted the present study to evaluate the specific roles of CCN2 with tissues, cells and animal models of UBC. The expression profiles of CCN2 in UBC clinical specimens were characterized, and influences of CCN2 on the biological behaviors as well as sensitivity to chemotherapy of UBC cells were also analyzed. This study is likely to aid in further elucidating the functions and mechanisms of ECM in human UBC and may provide a reference for clinical treatment.

## RESULTS

### CCN2 is overexpressed in UBC specimens and associated with unfavorable pathologic features

**Figure 1:**
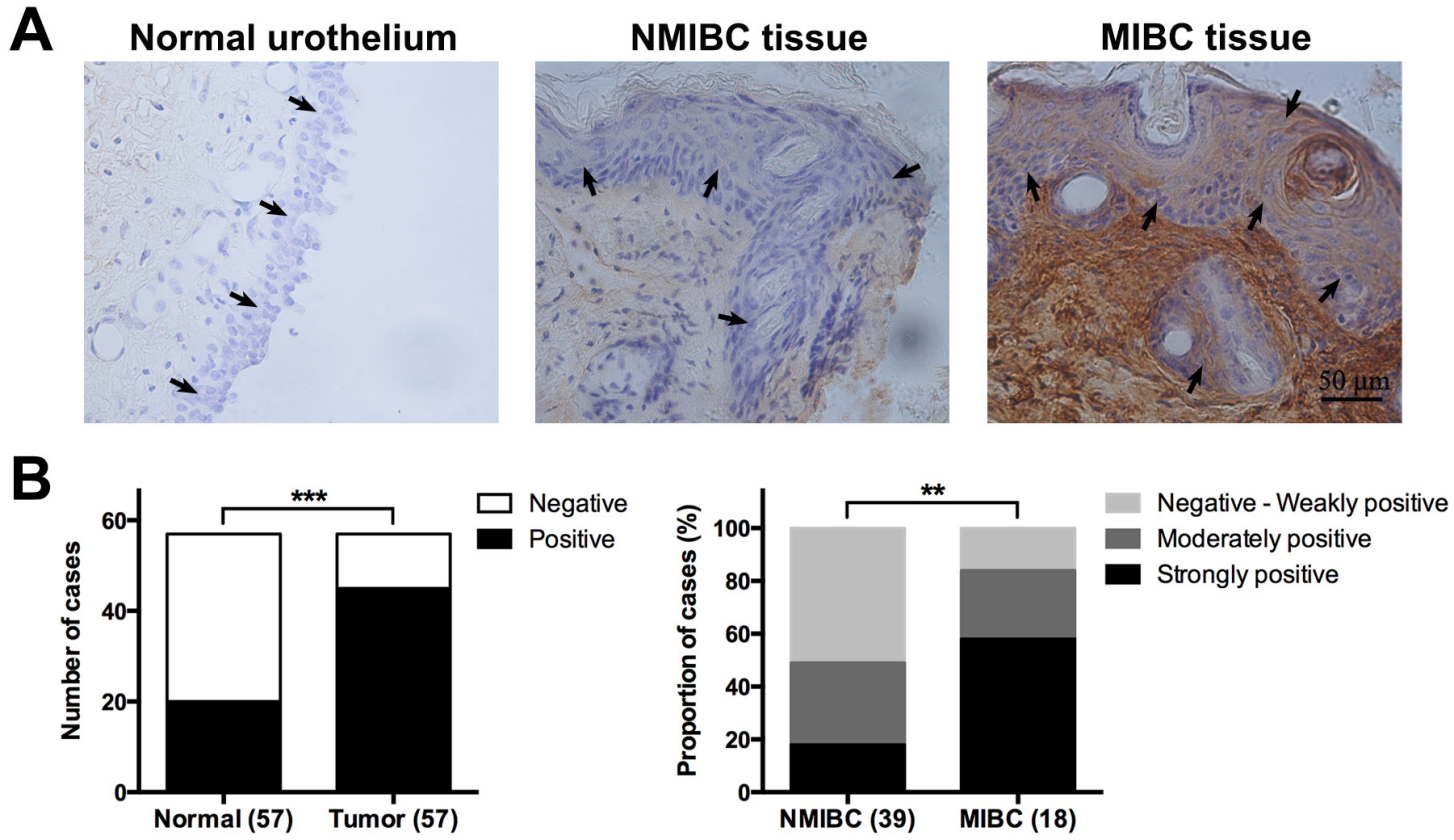
CCN2 expression in tumor/non-tumor urothelial tissues **(A)** Representative images of CCN2 staining for normal urothelium, NMIBC and MIBC tissues, respectively; bars: 50 μm. **(B)** Comparison in distribution of CCN2 staining status (positive or negative) between normal urothelial and UBC tissues (left panel) and that in distribution of CCN2 immunoreactivity levels (negative to weakly positive, moderately positive, strongly positive) between NMIBC and MIBC tissues (right panel). Data presented as number or percentage of patients. **, *P* < 0.01; ***, *P* < 0.001.

Expression of CCN2 was assessed by immunohistochemistry (IHC) assay, in 57 pairs of tumor/non-tumor urothelial samples obtained from UBC patients. To evaluate CCN2 staining, we mainly focused on urothelial/tumor cells and local surrounding areas. As shown in Figure 1A, CCN2 is mainly distributed in the extracellular matrix, as well as in cytoplasm of bladder cancer cells. It was common that UBC tissue showed increased CCN2 expression compared with normal urothelium. Furthermore, MIBC specimens usually displayed higher expression densities of CCN2 than NMIBC phenotype did. The index of CCN2 immunoreactivity was also introduced for semi-quantitative assessment, significantly more positive staining was observed in UBC specimens and higher proportion of invasive tumors (MIBC), rather than superficial lesions (NMIBC), was strongly positive (Figure 1B).

We further estimated the association of CCN2 expression with clinicopathologic parameters (Table 1). Patients with different CCN2 expression levels had no significant difference in age, gender or prior recurrence history. However, moderately or strongly positive expression of CCN2 were more likely to accompany with advanced stage and high grade in UBC lesions, further indicating that CCN2 might contribute to malignant features of UBC. We also analyzed the influence of CCN2 expression on disease recurrence or progression in 46 patients who received bladder-preserving surgery. During the follow-up, more recurrence occurred in cases with higher CCN2 expression, but the difference was not statistically significant. There were two cases of progression and both were among patients with higher CCN2 expression (Table 1). Our result implies that UBC patients with high CCN2 expression may have poor oncologic outcomes.

**Table 1:** Clinicopathologic characteristics and oncologic results of patients with UBC and CCN2 expression

Variable	Patients, n (%)	CCN2 expression		P-value
		(−)∼(+)	(2+)∼(3+)	
Age (y)				0.472
<60	24 (42.1)	11	13	
≥60	33 (57.9)	12	21	
Gender				0.501
Male	40 (70.2)	15	25	
Female	17 (29.8)	8	9	
Primary				0.209
Yes	34 (59.6)	16	18	
No	23 (40.4)	7	16	
Stage				0.013
Ta-T1	39 (68.4)	20	19	
T2-T4	18 (31.6)	3	15	
Grade*				
Low	31 (54.4)	17	14	0.015
High	26 (45.6)	6	20	
Recurrence^#^				0.306
Yes	10 (21.7)	3	7	
No	36 (78.3)	18	18	
Progression^#^				NA
Yes	2 (4.3)	0	2	
No	44 (95.7)	21	23	

* Histologic grading of UBC specimens was performed according to 2004 WHO classification: papillary urothelial neoplasm of low malignant potential (PUNLMP) is defined as a papillary urothelial tumor that resembles the exophytic urothelial papilloma, but with increased cellular proliferation exceeding the thickness of normal urothelium; low grade urothelial carcinoma, consisting of slender papillae with frequent branching and variation in nuclear polarity, shows fronds with recognizable variation in architecture and cytology, the nuclei show enlargement and irregularity with vesicular chromatin, nucleoli are often present and mitotic figures may occur at any level; high grade urothelial carcinoma is characterized as the cells lining the papillary fronds that show obviously disordered arrangement with cytologic atypia, the papillae are frequently fused, nuclei are pleomorphic with prominent nucleoli and altered polarity, mitotic figures are frequent and the thickness of the urothelium varies considerably.

Low, PUNLMP or low grade urothelial carcinoma; high, high grade urothelial carcinoma.

^#^ Influences of CCN2 expression on oncologic results were analyzed in 46 patients who received bladder-preserving surgery; recurrence was defined as any evidence of lesions in the bladder at least 3 months after complete removal of tumors; progression was defined as recurrence with increase to pT2-4 stage (for NMIBC) or higher TNM stage (for MIBC).

### Down-regulation of CCN2 inhibits proliferation of UBC cells

**Figure 2:**
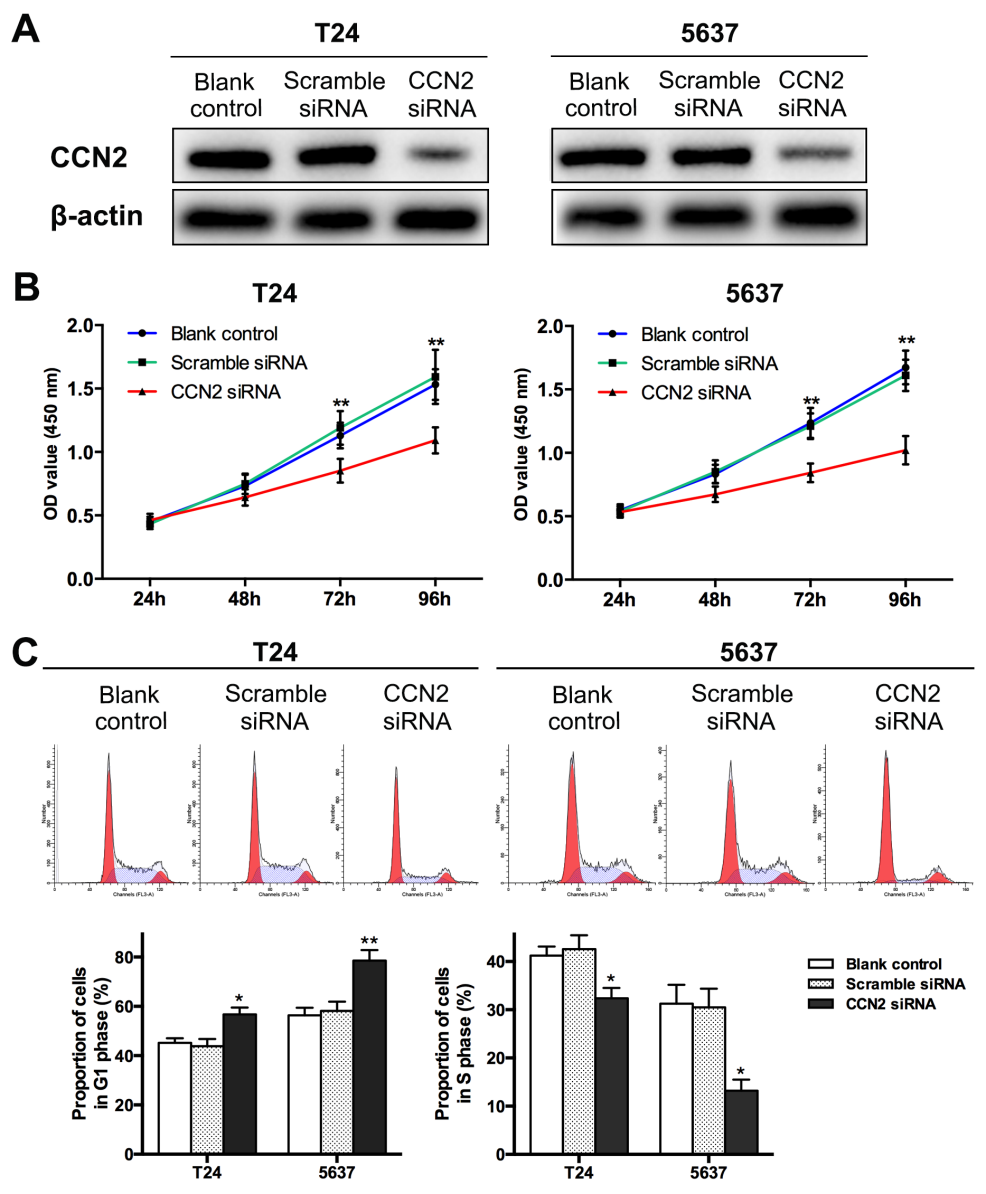
Down-regulation of CCN2 suppresses proliferation of UBC cells **(A)** T24 and 5637 cells were transfected with CCN2 siRNA; cells without transfection and those transfected with scramble siRNA served as blank and negative control, respectively; CCN2 expression was detected by western blotting assay around 48-72 h after transfection; β-actin was used as a loading control. **(B)** At indicated time points (24, 48, 72, 96 h after seeding, namely 12, 36, 60, 84 h after transfection, respectively), CCK-8 assays were performed to evaluate the proliferation capacities of UBC cells. **(C)** Representative cell cycle distribution of each group at 48 h after transfection as detected by FCM (upper panel) andcomparison of proportions of G1-phase or S-phase cells (lower panel). Data presented as mean ± SEM. *, *P* < 0.05; **, *P* < 0.01.

Functional studies on CCN2 was performed in two UBC cell lines T24 and 5637. The knockdown of protein was carried out by transfection with CCN2 small interfering RNA (siRNA) and then verified by western blotting assays around 48-72 h after transfection(Figure 2A). CCK-8 assay was employed to estimate the effect of CCN2 in cell proliferation. UBC cells without transfection or those transfected with scramble siRNA kept healthy growth, whereas silencing of CCN2 remarkably decelerated the proliferation (Figure 2B). We also performed flow cytometry (FCM) to analyze the changes of cell cycle in cells collected at 48 h after transfection. Consistently, knockdown of CCN2 significantly decreased the proportion of S-phase cells but increased G1-phase cells (Figure 2C). These results suggested that down-regulation of CCN2 was able to inhibit the proliferation of UBC cells.

### Down-regulation of CCN2 suppresses migration and invasion of UBC cells

**Figure 3:**
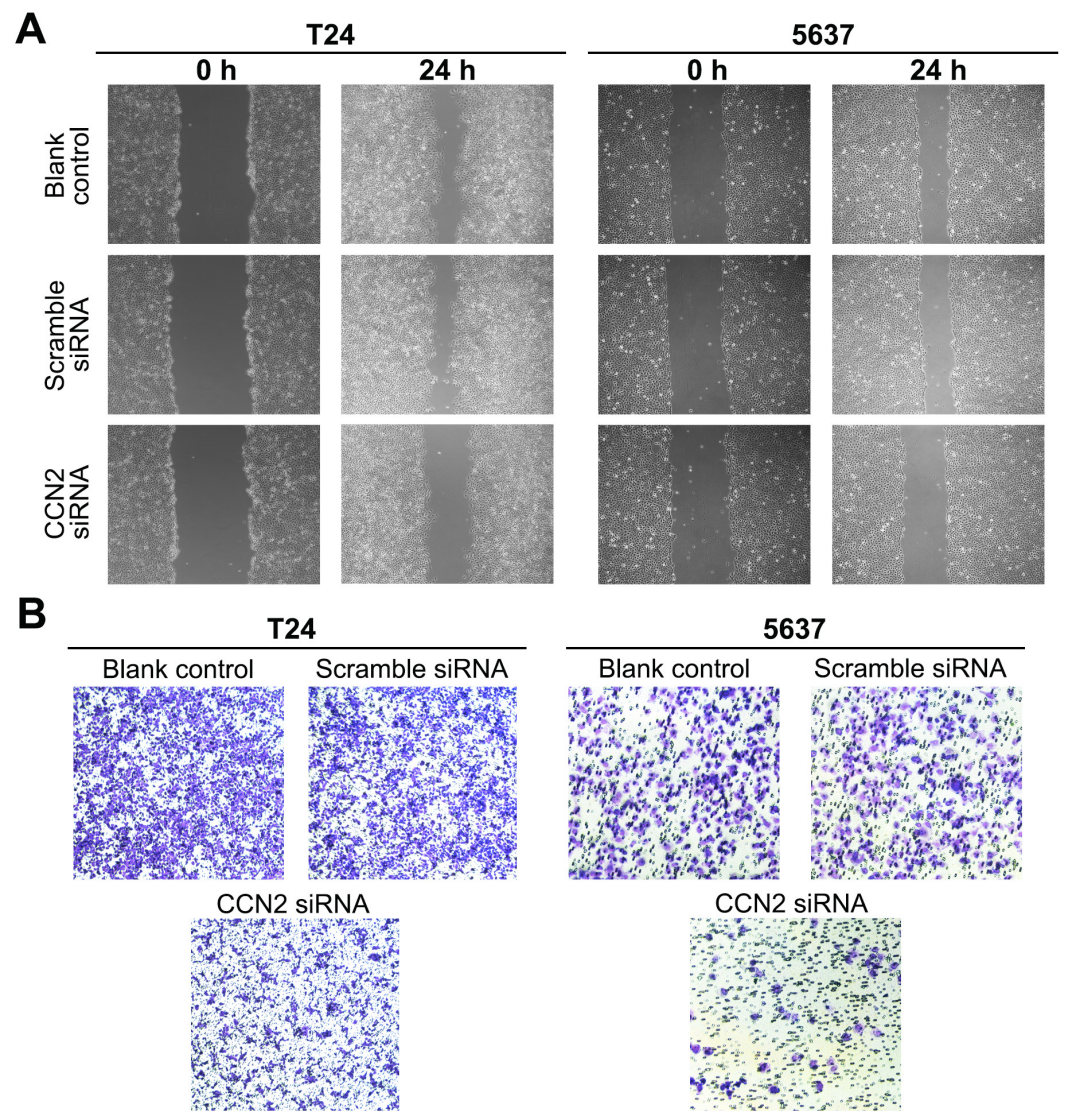
Down-regulation of CCN2 inhibits migration and invasion of UBC cells **(A)** T24 and 5637 cells were either without transfection (blank control) or transfected with CCN2 or scramble siRNA, and then subjected to wound healing assay to determine the migration capacity at 24 h following scarification. **(B)** Transwell assay was carried out to evaluate the invasion capacity of UBC cells.

Metastasis is regarded as the major cause of mortality in UBC patients and the biological process requires migration of cancer cells and invasion into surrounding and distant tissues. We performed the wound healing assay to determine the cell migration. As indicated in Figure 3A, CCN2-siliencing UBC cells filled the wound more slowly than other control groups did. The ability of cell invasion was evaluated using the transwell assay, and remarkably fewer CCN2-siliencing cells penetrated through matrigel and reached the lower transwell chamber (Figure 3B). Above results demonstrate that knockdown of CCN2 impairs migration and invasive abilities of UBC cells.

### Targeting of CCN2 prevents UBC development in xneograft

**Figure 4:**
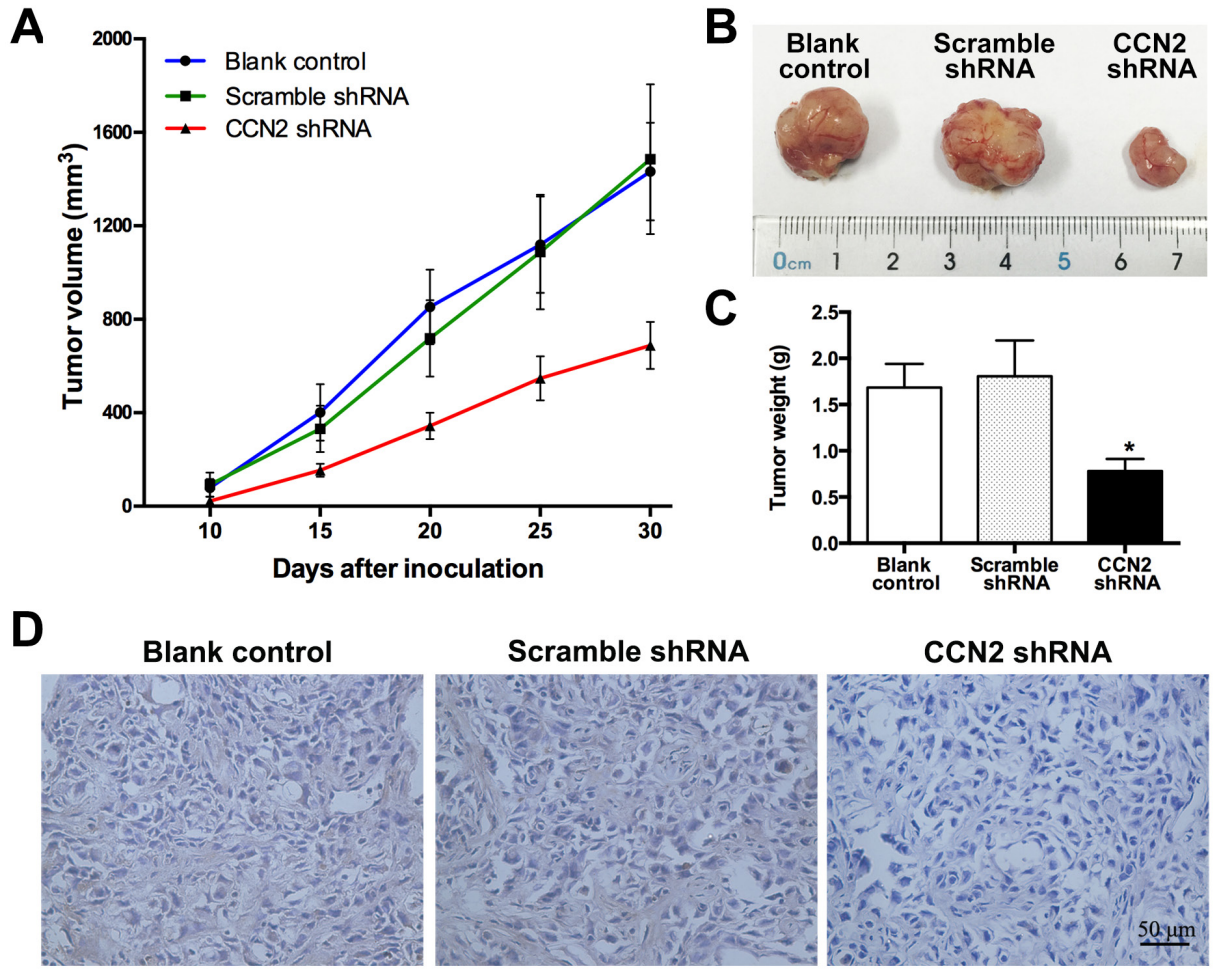
Targeting of CCN2 attenuates xenograft tumor growth *in vivo* **(A)** Tumor growth curves for xenograft tumors of un-transfected, scramble shRNA-transfected and CCN2 knockdown T24 cells, respectively; sizes of tumors were measured every five days since 10 days after inoculation and the whole duration of *in vivo* tumor growth was 30 days. **(B)** Representative photographs of xenograft tumors at the end of the experiment (the 30^th^ day). **(C)** Tumor weights of all groups. **(D)** Representative images of xenograft tumor sections with CCN2 staining; bars: 50 μm. Data presented as mean ± SEM; *, *P* < 0.05.

To investigate *in vivo* effect of CCN2 on tumor growth, T24 cells stably transfected with CCN2 or scramble short hairpin RNA (shRNA), or parental cells were injected subcutaneously to construct the xenograft models. Compared with the control groups, inhibition of CCN2 obviously decelerated the growth of T24 xenografts (Figure 4A) and resulted in significant reduction in tumor size (Figure 4B) and weight (Figure 4C) at the termination of experiment. As expected, IHC staining assays validated that CCN2 knockdown by shRNA effectively attenuated the protein expression in xenografts (Figure 4D). Overall, targeting of CCN2 can inhibit development of UBC *in vivo*.

### Down-regulation of CCN2 improves sensitivity of UBC cells to MMC

**Figure 5:**
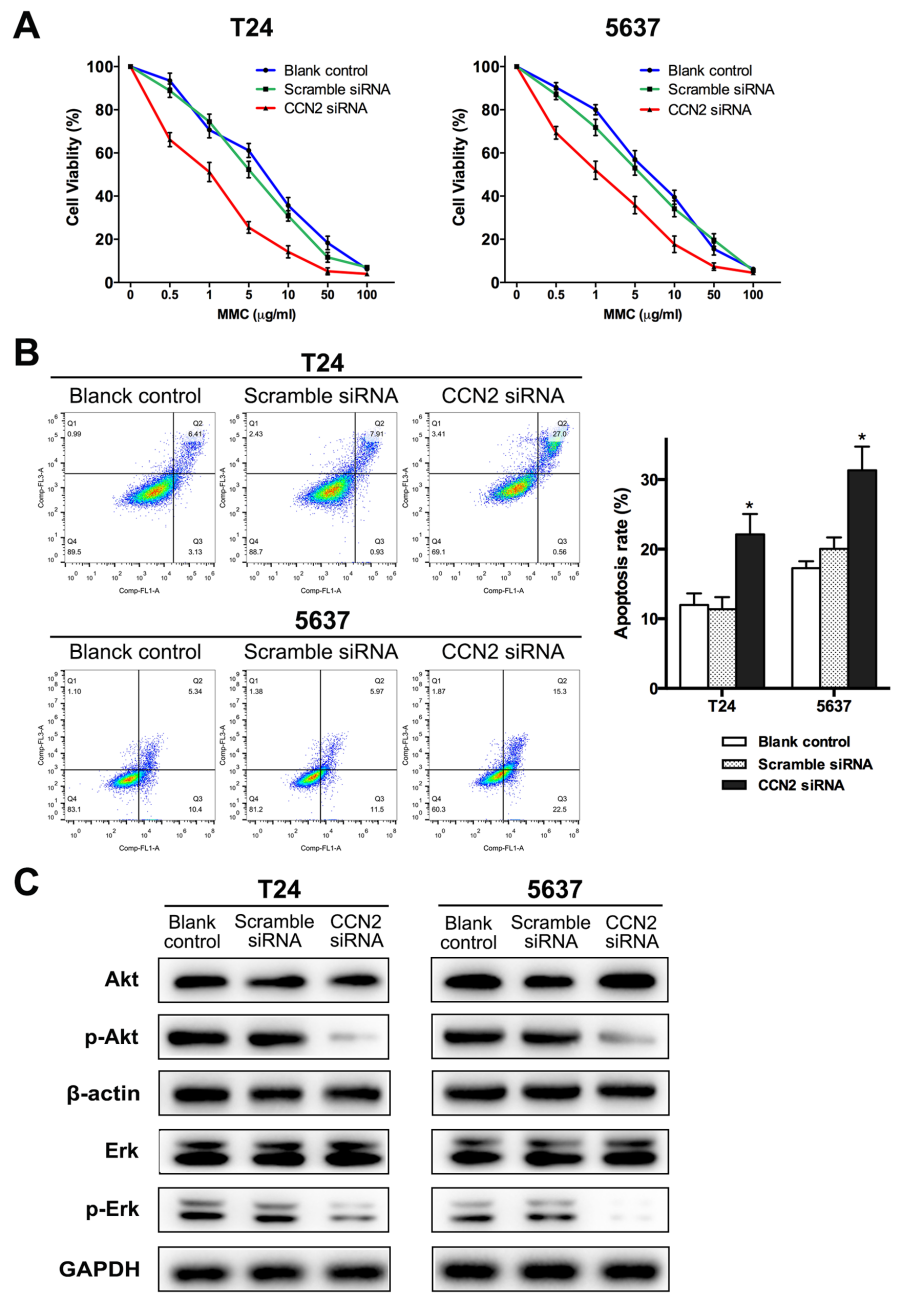
Down-regulation of CCN2 increases sensitivity of UBC cells to MMC via inhibiting activation Akt and Erk pathways **(A)** CCK-8 assay was performed to estimate survival rate curves of all groups after 24 h treatment with MMC at different concentrations. **(B)** Representative cell apoptosis distribution of each group as detected by FCM; region Q1, Q2, Q3 and Q4 shows dead, late apoptotic, early apoptotic and living cells, respectively; Q2 + Q3 regarded as apoptotic cells; all groups were treated with 100 μg/mL of MMC for 2 h after 48 h of transfection and then cultured in complete medium without MMC for 24 h, before apoptosis assessment. **(C)** Protein levels of CCN2, Akt, Erk, phospho-Akt and phospho-ERK for all groups were evaluated by western blotting assay, after MMC treatment (the same with MMC treatment protocol before apoptosis detection); β-actin or GAPDH was used as a loading control. Data presented as mean ± SEM; *, *P* < 0.05.

Mitomycin C (MMC) is a well-known chemotherapeutic agent for UBC. Here, we further evaluated the influence of CCN2 on efficacy of MMC. CCK-8 assay was carried out to determine the cytotoxicity of MMC in UBC cells. After cultured in media with different concentrations of MMC (0.1-100 μg/mL) for 24 h, knockdown of CCN2 resulted in significantly lower viable rates and half maximal inhibitory concentration (IC_50_) values of MMC in both T24 and 5637 cells (Figure 5A, Table 2). FCM analysis was employed to evaluate the cell apoptosis after MMC treatment (100 μg/mL for 2 h) and the total apoptotic rates for CCN2-siliencing UBC cells were higher than those of control groups (Figure 5B). Thus, down-regulation of CCN2 protein improves MMC chemo-sensitivity in UBC cells.

**Table 2:** IC50 values of MMC for each group of UBC cells

Group	IC_50_ (95% confidence interval) (μg/mL)	
	T24	5637
Blank control	5.97 (4.70-7.59)	6.26 (5.34-7.34)
Scramble siRNA	4.55 (3.84-5.41)	4.86 (3.96-5.96)
CCN2 siRNA	1.13 (0.94-1.36)	1.44 (1.14-1.82)

### Down-regulation of CCN2 suppresses activation of Akt and Erk pathways in UBC cells

To delineate the downstream mechanisms of CCN2-involved MMC chemo-sensitization, western blotting assay was performed to examine several key signaling cascades. After MMC treatment, there was no significant alteration in the levels of the majority of the signaling cascades, whether or not CCN-2 expression was inhibited. However, as compared to control groups, phosphorylated-Akt was decreased in both T24 and 5637 cells transfected with CCN2 siRNA. Similarly, knockdown of CCN2 also resulted in a decrease in Erk1/2 activation (Figure 5C). Herein, it can be speculated that Akt and Erk pathways mediate effect of CCN2 in UBC cells.

## DISCUSSION

UBC is a common urinary malignancy characterized by high possibilities of recurrence, progression and cancer-specific mortality. Although chemotherapy is the important adjuvant treatment after surgery, interior sensitivity or resistance to drugs becomes an obstacle to favorable oncologic outcomes. MMC, an antitumor antibiotic functioning through the formation of cross-linking with DNA, is a classical chemotherapeutic agent used alone or in combination with bacillus Calmette-Guérin as the intravesical therapy of NMIBC. The efficacy of MMC varied significantly among studies and recurrence and progression rate was as high as 46.4% and 9.4%, respectively [8, 9]. Here, for the first time, we focused on functions and mechanisms of CCN2, which was demonstrated overexpressed in UBC tissues. Silencing of CCN2 inhibited proliferation, migration and invasion of UBC cells. During MMC treatment, targeting of CCN2 improved cytotoxicity of MMC and promoted MMC-induced apoptosis, probably by inhibiting activation of proliferation-related Erk1/2 and survival-related Akt pathways. Our results suggest that CCN2 could protect chemo-resistance and it might be a novel target for improving therapeutic efficacy in UBC.

The expression of CCN2 in UBC tissues was rarely reported. Fang et al [7] revealed the down-regulation of CCN2 at T_a-1_ stage and up-regulation at T_1-2_ stage through microarray analysis. To expatiate the expression pattern, IHC staining of CCN2 was performed in clinical specimens. Since CCN2 is a stretch-related ECM protein expressed in bladder detrusor [10, 11], our observation was confined within the local area of urothelial/tumor cells and surrounding matrix. Compared with the normal urothelium, bladder tumors showed significantly higher expression of CCN2. Specifically, MIBC tissues usually exhibited moderately or strongly positive staining of CCN2 protein. High-grade tumors were also proven associated with remarkable expression of CCN2. The differential expression across malignant levels suggests a role for CCN2 in the development of UBC. Although no statistical significance was found regarding prior recurrence history, we can still find relatively more proportion of cases with higher CCN2 expression in recurrent tumors. Follow-up data also shows more recurrence and progression in tumors with high CCN2 expression. CCN2 might be a prognostic factor for UBC patients. Although the relatively small cohort and short-term follow-up duration limit the efficacy of statistics, further large-scale study with survival analysis could help validate this result.

CCN2 displays diverse roles in a variety of malignancies. In thyroid cancer and rhabdomyosarcoma, CCN2 accelerates tumor growth and inhibits cell apoptosis [12, 13]. In malignant melanoma and gastric cancer, CCN2 promotes cell invasion and migration [14, 15]. CCN2 also promotes angiogenesis in breast cancer and osteosarcoma [16, 17]. By contrast, CCN2 functions as tumor suppressors in a few circumstances. For example, CCN2 inhibits metastasis and invasion in lung adenocarcinoma and colorectal cancer, and CCN2 expression in cancer specimens correlates with relatively early-stage disease or better overall survival [18, 19]. CCN2 also has a negative effect on cell growth in oral squamous cell carcinoma [20]. In the present study, down-regulation of CCN2 expression suppressed proliferation of UBC cells and the inhibitory effect was validated in xenografts. Possible receptors of CCN2 include epidermal growth factor receptor [21], fibroblast growth factor receptor [22] and integrin families [23, 24]. After binding to these membrane proteins, CCN2 would accelerate cell proliferation by enhancing the effects of growth factors or triggering downstream signaling. Our study showed that silencing of CCN2 expression weakened capacities of migration and invasion in UBC cells. CCN2 can positively modulate expression of matrix metalloproteinases, which are critically involved in cancer metastasis [25, 26]. Besides, as an ECM member, CCN2 is capable of interacting with other ECM components, such as fibronectin and perlecan, thus facilitating cell adhesion and migration [24].

We have been investigating mechanisms of chemo-resistance in UBC and previously revealed that ECM protein, receptors and relevant molecules could be involved in resistance to MMC [27-29]. CCN2 was reported to confer chemo-resistance in several malignancies [6, 30, 31], however, no such study has been performed in UBC. Chen et al [32] found that administration of MMC brought into significant up-regulation of CCN2 in UBC cells. Accordingly, CCN2 expression is assumed to resist the chemotherapeutic effect. To some extent, this hypothesis is confirmed by our results that targeting of CCN2 improved cytotoxicity of MMC and promoted MMC-induced apoptosis. It was reported that CCN2 could induce expression of fibronectin [33], and MMC resistance could arise from fibronectin adhesion or expression of its receptors in UBC cells [27-29]. It can be deduced that CCN2 might result in chemo-resistance via fibronectin expression. In fact, both CCN2 and fibronectin belong to ECM ligands of integrin, an important receptor contributing to cancer development and drug resistance [34]. We observed that down-regulation of CCN2 in MMC-treated UBC cells deactivated survival-related Akt and proliferation-related Erk cascades, both of which are involved in integrin signaling [34]. Our pathway analysis agrees with the theory that tumor microenvironment may promote drug resistance via the interaction of cancer cells with ECM and subsequent activation of intracellular downstream cascades. Collectively, investigation of CCN2 will enrich the database of ECM-targeted strategies for cancer treatment.

In conclusion, our data reveal clinical significance and functional mechanisms of CCN2 as an oncogene in UBC. We demonstrate the high expression of CCN2 in UBC tissues as well as its association with unfavorable pathologic features. Targeting of CCN2 exerts inhibitory effects on tumor development *in vitro* and *in vivo*, and more importantly, suppression of CCN2 expression remarkably improves MMC sensitivity of UBC cells. These findings suggest CCN2 as a promising target for UBC treatment.

## MATERIALS AND METHODS

### Patients and tissue specimens

After approved by institutional ethics committee, 57 pairs of tumor/non-tumor urothelial specimens were collected from patients at our department from 2014 to 2016. There were 34, 12 and 11 patients receiving TURBT, partial cystectomy and radical cystectomy, respectively. The specimens were fixed in 10% formaldehyde solution and embedded in paraffin for further IHC staining. The needed clinicopathologic data were collected from electronic medical records to make correlation analysis regarding CCN2 expression. Follow-up data were collected in the 46 patients who received bladder-preserving surgery (TURBT or partial cystectomy). Recurrence was defined as any evidence of lesions in the bladder at least 3 months after complete removal of tumors, and progression was defined as recurrence with increase to pT2-4 stage (for NMIBC) or higher TNM stage (for MIBC).

### IHC staining

Paraffin-embedded tissues from patients or xenografts of mice were cut into 5-μm sections and then deparaffinized and rehydrated. The slides were incubated with antibody against CCN2 (1:100, Santa Cruz Biotechnology, Santa Cruz, CA, USA) overnight at 4 °C and normal IgG instead of primary antibodies was as negative control. Then the slides were washed with PBS and incubated at room temperature for 2 h using Envision kit (DAKO, Carpinteria, CA, USA). Two independent observers made an assessment of sections based on the intensity and proportion of staining. Briefly, staining intensity was classified into four grades (negative, weak, moderate, strong; scored as 0, 1, 2 and 3, respectively) and staining proportion was into four levels (≤25%, 26-50%, 51-75%, >75%; scored as 0, 1, 2 and 3, respectively). The sum score of intensity and proportion scores was calculated, and CCN2 immunoreactivity was defined as follows: negative, weakly positive, moderately positive, and strongly positive (sum score: 0, 1-2, 3-4 and 5-6, respectively).

### Cell culture

Two human UBC cell lines T24 and 5637 were obtained from Type Chinese Academy of Science (Shanghai, China) and RPMI 1640 medium supplemented with 10% fetal bovine serum (FBS, Gibco, Carlsbad, CA, USA) was used for cell culture, under a humidified atmosphere of 5% CO_2_ and 95% air at 37 °C.

### siRNA transfection

Experimentally verified CCN2 and scramble siRNA were chemically synthesized by GenePharma Co., Ltd. (Shanghai, China) and the sequences are as follows:

**Table:** 

CCN2 siRNA	sense:	5’-UCAAAGAUGUCAUUGUCUCCG-3’
	antisense:	5’-GAGACAAUGACAUCUUUGAAU-3’
Scramble siRNA	sense:	5’-UUCUCCGAACGUGUCACGUTT-3’
	antisense:	5’-ACGUGACACGUUCGGAGAATT-3’

Transfection of siRNA was performed using lipofectamine 2000 reagent (Invitrogen, Carlsbad, CA, USA) as the manufacturer’s instruction.

### Cell proliferation assay

Approximately 2 × 10^3^ UBC cells were plated in each well of 96-well plates and transfection was performed after overnight incubation. At indicated time points after seeding, the numbers of viable cells were assessed with Cell Counting Kit-8 (CCK-8), according to the manufacturer’s protocol (Dojindo, Kunamoto, Japan). Absorbance was measured at 450 nm on a spectrophotometer and normalized by subtracting the mean of blank well values. These experiments were performed in sextuplicate.

### Cell migration assay

Cell migration activity was evaluated by wound healing assay. In 6-well plates, transfection of CCN2 or scramble siRNA was conducted in UBC cells. Upon reaching confluence, the cell monolayer was scraped straightly with a micropipette tip and washed with phosphate-buffered saline (PBS) to remove cell debris. After incubated in serum-free medium for 24 h, gap distances of scraped monolayer after wounding were measured under light microscope, at indicated time points.

### Cell invasion assay

Cell invasion was measured using 24-well transwell chamber with 8-μm-pore insert coated with matrigel (Corning, NY, USA). In serum-free medium, about 5 × 10^4^ re-suspended transfected UBC cells were plated in the upper compartment of chamber. Complete medium as chemo-attractant was added into the lower chamber. After incubated for 24 h, remaining cells on the upper side of inserts were gently scraped off and invaded cells on the lower surface were fixed in 4 % paraformaldehyde and stained with 0.1 % crystal violet. For each insert, three random fields were selected to count numbers of invaded cells under light microscope.

### *In vitro* MMC sensitivity assay

CCK-8 assay was carried out to assess the effect of MMC on cell viability. Transfected UBC cells were seeded onto 96-well plates, with 0.1-100 μg/mL MMC added in culture for 24 h. Cell survival rate was calculated as a percentage of the corresponding control (0 μg/mL MMC) absorbance. These experiments were performed in sextuplicate and IC_50_ values were calculated by using Graphpad Prism 6 software. Besides, FCM was employed to evaluate cell apoptosis. UBC cells seeded onto 6-well plates were transfected with CCN2 or scramble siRNA. At 48 h, viable cells were harvested, seeded and subsequently treated with 100 μg/mL of MMC (Sigma) for 2 h. Then UBC cells were further cultured in complete medium without MMC for 24 h, before apoptosis assessment. These experiments were performed three times.

### Flow cytometry (FCM)

We performed FCM to analyze cell cycle and apoptosis. In 6-well plates, UBC cells were transfected with CCN2 or scramble siRNA and collected at 48 h. After fixed with 70% ice ethanol overnight at 4 °C, the centrifuged cells were then stained with propidium iodide/RNase buffer (BD Biosciences, San Jose, CA, USA), according to the manufacturer’s instruction. The data were collected and analyzed on FACScalibur flow cytometer with the CellQuest software (BD Biosciences). These experiments were performed a minimum of three times. To make cell apoptosis analysis, double staining of fluorescein isothiocyanate (FITC) labeled annexin-V and propidium iodide was performed with Apoptosis Detection kit (Dojindo, Kunamoto, Japan) in MMC-treated UBC cells, according to the manufacturer’s protocol. The stained cell suspension was subjected to FCM analysis, and UBC cells were discriminated into living, dead, early apoptotic and late apoptotic cells, with distribution determined by the FlowJo 7.6 software (Treestar, Ashland, OR, USA).

### Protein extraction and western blotting assay

Transfected or MMC-treated UBC cells were washed in cold PBS twice and lysed on ice with RIPA buffer containing protease and phosphatase inhibitors (Thermo Fisher Scientific, Franklin, MA, USA). After concentration measurement (BCA Protein Assay Kit, Beyotime, China), 20 mg of denatured protein were loaded on SDS-PAGE gel and transferred onto polyvinylidene diﬂuoride membranes (Millipore, Billerica, MA, USA). Then membranes were blocked for 2 h in Tris buffered saline with 0.1% tween (TBST) containing 5% bovine serum albumin (Sigma), and subsequently incubated overnight at 4 °C with primary antibodies against CCN2 (Santa Cruz), Akt, phospho-Akt, ERK, phospho-ERK, GAPDH or β-actin (Cell Signaling Technology, Beverly, USA) at recommended dilutions. After complete washing in TBST, membranes were incubated with the corresponding horseradish peroxidase-conjugated secondary antibodies at room temperature for 1 h and visualized with ECL substrates (Millipore). GAPDH and β-actin were used as a loading control.

### shRNA-mediated CCN2 knockdown

The shRNA vectors were constructed by GenePharma Co., Ltd. CCN2 and scramble control shRNAs were generated using the following sequences, respectively:

CCN2 shRNA duplexes:5’-CACCGCTGACCTGGAAGAGAACATTCGAAAATGTTCTCTTCCAGGTCAGC-3’5’-AAAAGCTGACCTGGAAGAGAACATTTTCGAATGTTCTCTTCCAGGTCAGC-3’

scramble shRNA duplexes:5’-CACCAAACGTGACACGTTCGGAGAACGAATTCTCCGAACGTGTCACGTTT-3’5’-AAAAAAACGTGACACGTTCGGAGAATTCGTTCTCCGAACGTGTCACGTTT-3’

The amplicons were cloned into pLKO.1-puro plasmid (Sigma, St. Louis, MO, USA) to construct CCN2 shRNA vector and its scramble control, respectively. Transfection of plasmids in T24 cells was carried out using lipofectamine 2000, and stably transfected cells were selected by culturing in the presence of 2 μg/ml puromycin (Sigma) for more than two weeks.

### Animal experiment

Animal experiment in the present study was approved by the insititutional animal experimentation committee. Fifteen 4-week-old female BALB/c nude mice were randomized into three equal groups, which were then subcutaneously inoculated with un-transfected, scramble shRNA-transfected and CCN2 knockdown T24 cells, respectively. The size of tumors and body weight of mice were measured every five day, and tumor volume was calculated with the formula length × width^2^ ×0.5. At the end of experiments, all mice were sacrificed and tumors were excised and weighed. Tumor tissues were obtained for further analyses.

### Statistical analysis

SPSS 16.0 software (SPSS Inc., Chicago, IL, USA) was used for statistical analyses in this study. The one-way ANOVA and the χ2 test (or Fisher test) were employed to compare quantitative data and categorical data, respectively. In all tests, *P* < 0.05 was considered statistically significant.

## References

[1] Torre LA, Bray F, Siegel RL, Ferlay J, Lortet-Tieulent J, Jemal A (2015). Global cancer statistics, 2012. CA Cancer J Clin.

[2] Siegel RL, Miller KD, Jemal A (2017). Cancer statistics, 2017. CA Cancer J Clin.

[3] Köberle B, Piee-Staffa A (2012). The Molecular Basis of Cisplatin Resistance in Bladder Cancer Cells. INTECH Open Access Publisher.

[4] Krishna R, Mayer LD (2000). Multidrug resistance (MDR) in cancer. Mechanisms, reversal using modulators of MDR and the role of MDR modulators in influencing the pharmacokinetics of anticancer drugs. Eur J Pharm Sci.

[5] Zuo GW, Kohls CD, He BC, Chen L, Zhang W, Shi Q, Zhang BQ, Kang Q, Luo J, Luo X, Wagner ER, Kim SH, Restegar F (2010). The CCN proteins: important signaling mediators in stem cell differentiation and tumorigenesis. Histol Histopathol.

[6] Tsai HC, Huang CY, Su HL, Tang CH (2014). CCN2 enhances resistance to cisplatin-mediating cell apoptosis in human osteosarcoma. PLoS One.

[7] Fang ZQ, Zang WD, Chen R, Ye BW, Wang XW, Yi SH, Chen W, He F, Ye G (2013). Gene expression profile and enrichment pathways in different stages of bladder cancer. Genet Mol Res.

[8] Boöhle A, Jocham D, Bock PR (2003). Intravesical bacillus Calmette-Guerin versus mitomycin C for superficial bladder cancer: a formal meta-analysis of comparative studies on recurrence and toxicity. J Urol.

[9] Boöhle A, Bock PR (2004). Intravesical bacille Calmette-Guérin versus mitomycin C in superficial bladder cancer: formal meta-analysis of comparative studies on tumor progression. Urology.

[10] Chaqour B, Whitbeck C, Han JS, Macarak E, Horan P, Chichester P, Levin R (2002). Cyr61 and CTGF are molecular markers of bladder wall remodeling after outlet obstruction. Am J Physiol Endocrinol Metab.

[11] Ramachandran A, Gong EM, Pelton K, Ranpura SA, Mulone M, Seth A, Gomez P, Adam RM (2011). FosB regulates stretch-induced expression of extracellular matrix proteins in smooth muscle. Am J Pathol.

[12] Cui L, Zhang Q, Mao Z, Chen J, Wang X, Qu J, Zhang J, Jin D (2011). CTGF is overexpressed in papillary thyroid carcinoma and promotes the growth of papillary thyroid cancer cells. Tumour Biol.

[13] Croci S, Landuzzi L, Astolfi A, Nicoletti G, Rosolen A, Sartori F, Follo MY, Oliver N, De Giovanni C, Nanni P, Lollini PL (2004). Inhibition of connective tissue growth factor (CTGF/CCN2) expression decreases the survival and myogenic differentiation of human rhabdomyosarcoma cells. Cancer Res.

[14] Braig S, Wallner S, Junglas B, Fuchshofer R, Bosserhoff AK (2011). CTGF is overexpressed in malignant melanoma and promotes cell invasion and migration. Br J Cancer.

[15] Mao Z, Ma X, Rong Y, Cui L, Wang X, Wu W, Zhang J, Jin D (2011). Connective tissue growth factor enhances the migration of gastric cancer through downregulation of E-cadherin via the NF-κB pathway. Cancer Sci.

[16] Wang LH, Tsai HC, Cheng YC, Lin CY, Huang YL, Tsai CH, Xu GH, Wang SW, Fong YC, Tang CH (2017). CTGF promotes osteosarcoma angiogenesis by regulating miR-543/angiopoietin 2 signaling. Cancer Lett.

[17] Chien W, O’Kelly J, Lu D, Leiter A, Sohn J, Yin D, Karlan B, Vadgama J, Lyons KM, Koeffler HP (2011). Expression of connective tissue growth factor (CTGF/CCN2) in breast cancer cells is associated with increased migration and angiogenesis. Int J Oncol.

[18] Chang CC, Shih JY, Jeng YM, Su JL, Lin BZ, Chen ST, Chau YP, Yang PC, Kuo ML (2004). Connective tissue growth factor and its role in lung adenocarcinoma invasion and metastasis. J Natl Cancer Inst.

[19] Lin BR, Chang CC, Che TF, Chen ST, Chen RJ, Yang CY, Jeng YM, Liang JT, Lee PH, Chang KJ, Chau YP, Kuo ML (2005). Connective tissue growth factor inhibits metastasis and acts as an independent prognostic marker in colorectal cancer. Gastroenterology.

[20] Moritani NH, Kubota S, Nishida T, Kawaki H, Kondo S, Sugahara T, Takigawa M (2003). Suppressive effect of overexpressed connective tissue growth factor on tumor cell growth in a human oral squamous cell carcinoma-derived cell line. Cancer Lett.

[21] Rayego-Mateos S, Rodrigues-Diez R, Morgado-Pascual JL, Rodrigues Diez RR, Mas S, Lavoz C, Alique M, Pato J, Keri G, Ortiz A, Egido J, Ruiz-Ortega M (2013). Connective tissue growth factor is a new ligand of epidermal growth factor receptor. J Mol Cell Biol.

[22] Aoyama E, Kubota S, Takigawa M (2012). CCN2/CTGF binds to fibroblast growth factor receptor 2 and modulates its signaling. FEBS Lett.

[23] Arnott JA, Lambi AG, Mundy C, Hendesi H, Pixley RA, Owen TA, Safadi FF, Popoff SN (2011). The role of connective tissue growth factor (CTGF/CCN2) in skeletogenesis. Crit Rev Eukaryot Gene Expr.

[24] Aguiar DP, de Farias GC, de Sousa EB (2014). de Mattos Coelho-Aguiar J, Lobo JC, Casado PL, Duarte ME, Abreu JG Jr. New strategy to control cell migration and metastasis regulated by CCN2/CTGF. Cancer Cell Int.

[25] Chintala H, Liu H, Parmar R, Kamalska M, Kim YJ, Lovett D, Grant MB, Chaqour B (2012). Connective tissue growth factor regulates retinal neovascularization through p53 protein-dependent transactivation of the matrix metalloproteinase (MMP)-2 gene. J Biol Chem.

[26] Tsai HC, Su HL, Huang CY, Fong YC, Hsu CJ, Tang CH (2014). CTGF increases matrix metalloproteinases expression and subsequently promotes tumor metastasis in human osteosarcoma through down-regulating miR-519d. Oncotarget.

[27] Pan CW, Shen ZJ, Wu TT, Tang XY, Wang M, Sun J, Shao Y (2009). Cell adhesion to fibronectin induces mitomycin C resistance in bladder cancer cells. BJU Int.

[28] Zhang CJ, Shen ZJ, Pan CW, Zhong S, Li T, Zhang MG (2012). Engagement of integrin beta1 induces resistance of bladder cancer cells to mitomycin-C. Urology.

[29] Xu T, Qin L, Zhu Z, Wang X, Liu Y, Fan Y, Zhong S, Wang X, Zhang X, Xia L, Zhang X, Xu C, Shen Z (2016). MicroRNA-31 functions as a tumor suppressor and increases sensitivity to mitomycin-C in urothelial bladder cancer by targeting integrin α5. Oncotarget.

[30] Wang MY, Chen PS, Prakash E, Hsu HC, Huang HY, Lin MT, Chang KJ, Kuo ML (2009). Connective tissue growth factor confers drug resistance in breast cancer through concomitant up-regulation of Bcl-xL and cIAP1. Cancer Res.

[31] Yin D, Chen W, O’Kelly J, Lu D, Ham M, Doan NB, Xie D, Wang C, Vadgama J, Said JW, Black KL, Koeffler HP (2010). Connective tissue growth factor associated with oncogenic activities and drug resistance in glioblastoma multiforme. Int J Cancer.

[32] Chen SK, Chung CA, Cheng YC, Huang CJ, Chen WY, Ruaan RC, Li C, Tsao CW, Hu WW, Chien CC (2014). Toll-like receptor 6 and connective tissue growth factor are significantly upregulated in mitomycin-C-treated urothelial carcinoma cells under hydrostatic pressure stimulation. Genet Test Mol Biomarkers.

[33] Mendes FA, Coelho Aguiar JM, Kahn SA, Reis AH, Dubois LG, Romao LF, Ferreira LS, Chneiweiss H, Moura Neto V, Abreu JG (2015). Connective-tissue growth factor (CTGF/CCN2) induces astrogenesis and fibronectin expression of embryonic neural cells in vitro. PLoS One.

[34] Aoudjit F, Vuori K (2012). Integrin signaling in cancer cell survival and chemoresistance. Chemother Res Pract.

